# Association of the high-sensitive cardiac troponin T levels and long-term mortality in patients with acute aortic dissection type A

**DOI:** 10.34172/jcvtr.2023.31624

**Published:** 2023-06-29

**Authors:** Yaser Jenab, Seyed-Hossein Ahmadi-Tafti, Tahereh Davarpasand, Arash Jalali, Hamid Khederlou

**Affiliations:** ^1^Tehran Heart Center, Tehran University of Medical Sciences, Tehran, Iran; ^2^Department of Cardiac Surgery, Tehran University of Medical Sciences, Tehran, Iran

**Keywords:** Cardiac Troponin, Aorta, Aortic Dissection, Mortality

## Abstract

**Introduction:**

Acute aortic dissection type A is a life-threatening cardiovascular emergency necessitating rapid diagnosis and treatment. We sought a new prognostic tool with cardiac biomarkers and simple inflammatory factors.

**Methods:**

from 2003 to 2014, 50 patients with documented acute aortic dissection type A were entered to this study. These patients were followed up until December 2020; within median follow up of 93.6 months. The patients were evaluated on the association of the baseline characteristics, first laboratory investigation, echocardiographic findings, surgical approach, and long-term mortality.

**Results:**

Total number of mortality during the follow up was 29 (58%) patients, which was significantly higher in medical group (89.4% vs 38.7%, *P* value=0.001). Multivariable analysis showed only an increase in hs-cTnT levels was suggested as a predictor of mortality (95% CI: 1.06–1.38; HR=1.21; *P*=0.005), so that for every 100 units increase, patients were 21% more likely to have mortality in long term. Also, performing surgical treatment for aortic dissection was determined as the independent predictor of surviving, so that death was 74.6% less than those who received medical treatment (95% CI: 0.13–0.58; HR=0.27; *P*=0.001).

**Conclusion:**

hs-cTnT is a potential predictor of mortality in patients with acute aortic dissection type A.

## Introduction

There are two types of aortic dissection; type A and type B. type A, if the aortic dissection occurs in the ascending aorta and type B, if aortic dissection originates in the descending aorta.^1^ Acute aortic dissection (AAD) type A is a life-threatening cardiovascular emergency, with a mortality rate of 1%–2% per hour. In spite of the advances in the diagnosis of AAD through new diagnostic imaging modalities, AAD may prove fatal if not diagnosed timely and managed appropriately.^2^ Some laboratory tests such as red blood cell count, white blood cell count, cardiac troponin (cTn), and creatine kinase (CK) were recommended in the 2014 guidelines of the European Society of Cardiology (ESC) on the diagnosis and treatment of aortic disease.^3^

## Materials and Methods

**Figure 1:**
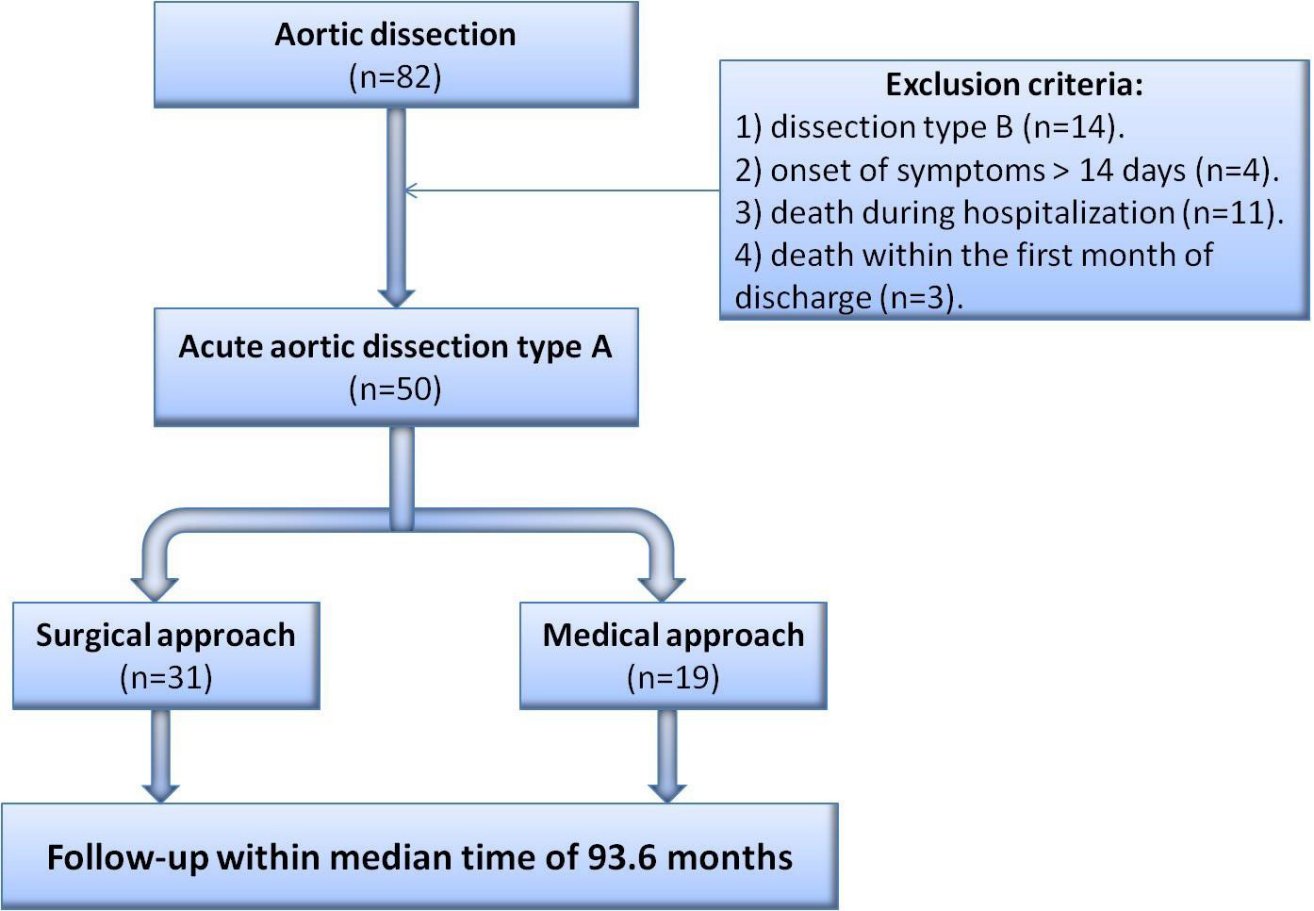
Sampling flowchart of the study for evaluation of the association between High-sensitive cardiac troponin T and long-term mortality in patients with acute aortic dissection type A

In a cross-sectional from September 2003 to September 2014, 82 patients were admitted at Tehran heart center with AAD. 50 patients who hadn’t exclusion criteria and they have inclusion criteria entered in this study. During hospitalization, 31 (62.0%) patients underwent surgery and the remaining patients (38%) were managed conservatively with close monitoring in the intensive care unit (ICU) to control blood pressure and heart rate with Sodium Nitroprusside and Esmolol because of being high risk for surgery according to responsible surgeon decision. Two groups (medical and surgical) evaluated in term of baseline characteristics, first laboratory investigation (white blood cell, platelet counts, serum creatinine, D-dimer, and hs-cTnT), echocardiographic findings. These patients were followed up until December 2020; within median follow up of 93.6 months (Figure 1).

###  Statistical analysis

The continuous variables are presented through means and standard deviations (SDs) or medians with interquartile ranges and were compared between the survivors and nonsurvivors using the Student *t*-test or the Mann–Whitney *U *test. The categorical variables are described as frequencies and percentages and were compared between the 2 mentioned groups using the χ^2^ test or the Fisher exact test. A backward logistic regression model was applied to find the multiple predictors of mortality. Variables with a *P* < 0.2 in the univariate analyses were candidate to enter the final model. The effect of the covariates on survival was reported as ORs with 95% CIs.

## Results

Patients who received medical treatment were significantly older than patients who underwent surgery. Also, patients with a history of CAD and CVA were more likely to receive medical treatment. Patients with at least moderate AI underwent more surgery (Table 1).

**Table 1:** Comparison of patients with surgical and medical treatment in terms of baseline characteristics, first laboratory investigation and echocardiographic findings

**Variable**	**Surgical group (n=31)**	**Medical group (n=19)**	***P*** **-value**
Gender (Male)	20 (64.50%)	9 (47.30%)	0.233
Age	53.06 ± 14.96	63.95 ± 16.15	0.019
Limb paralysis	3 (9.70%)	1 (5.30%)	0.998
syncope	4 (12.9%)	5 (26.30%)	0.273
SBP (mmHg)	138.90 ± 29.77	137.89 ± 24.21	0.901
Heart rate (Bits/min)	81.52 ± 17.70	89.05 ± 25.04	0.260
History of aortic disease	4 (12.90%)	5 (26.30%)	0273
History of CAD*	3 (9.70%)	8 (42.10%)	0.013
History of HTN*	18 (58.1%)	13 (68.40%)	0.464
History of CVA*	0 (0%)	3 (15.80%)	0.049
History of CKD*	1 (3.20%)	0 (0%)	0.998
hs Troponin T level (ng/dl)	21.49 (8.69-99.60)	20.52 (12.69-101.60)	0.984
D-dimer level (ng/dl)	1.70 (0.60-5.11)	4.86 (1.70-11.80)	0264
White blood cell count ( × 10³)	10.1 (8.70-13.01)	12.5 (9.70-16.02)	0.074
Hemoglobin (mg/dl)	13.03 ± 2.45	12.72 ± 1.93	0.644
Platelet count ( × 10³)	176.5 (133.20-225.40)	162 (115.20-237.10)	0.984
Neutrophil/Lymphocyte ratio	4.52 (2.54-8.48)	6.01 (4.18-9.44)	0.154
Creatinine (mg/dl)	1 (0.87-1.50)	1.03 (0.80-1.60)	0.952
Ejection fraction (%)	52.48 ± 9.61	54.37 ± 11.30	0.532
Moderate AI ≤	25 (83.30%)	11 (57.9%)	0.049
Pericardial effusion	13 (43.30%)	7 (36.8%)	0.652
Ascending aorta diameter (mm)	57.80 ± 12.97	51.35 ± 16.48	0.145

*SBP, Systolic blood pressure; CAD, Coronary artery disease; HTN, Hypertension; CVA, Cerebrovascular arrest; CKD, Chronic kidney disease; AI, Aortic insufficiency.

**Figure 2:**
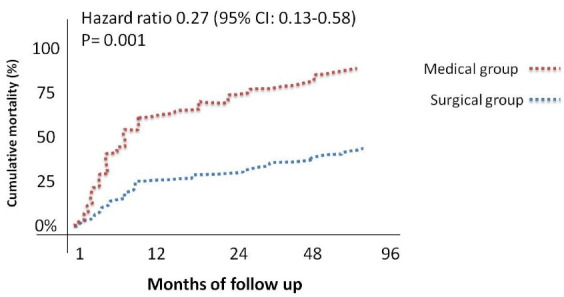
Kaplan-meier analysis of cumulative mortality among patients in the medical and surgical groups

Total number of mortality during the follow up was 29 (58%) patients, which was significantly higher in medical group (89.4% vs 38.7%, P-Value = 0.001) (Figure 2). Baseline characteristics of two groups (survivor and non-survivor) are depicted in Table 2. The mean age of patients was 57.2 ± 16.1 (27–84) years and more patients (58.0%) were male. The most frequent risk factor in patients with AAD type A was hypertension. A history of aortic disease or previous aortic root manipulation was positive in 9 (18.0%) patients: 1 (2.0%) patient with the Marfan syndrome, 1 (2.0%) patient with the Ehler-Danlos syndrome, 1 (2.0%) patient with aortic coarctation, 3 (6.0%) patients with a history of aortic root dilation, and 3 (6.0%) patients with a history of aortic valve replacement. There was no significant association between history of aortic disease and mortality. The mean left ventricular ejection fraction (LVEF) of the study population was 53.20% ± 10.30%. Moderate or severe aortic insufficiency (AI) and pericardial effusion were seen in 36 (73.5%) and 20 (40.8%) patients, respectively. In this study, elevated hs-cTnT ( ≥ 14 ng/L) was observed in 64.0% (n = 32) of patients. Above findings showed there were no statistically significant differences between the two groups in term of baseline characteristics and echocardiographic findings and mortality, however hs-cTnT and WBC were high in non-survivor group significantly.

**Table 2:** Association of baseline characteristics, first laboratory investigation, echocardiographic findings and surgical approach and survival of the patients

**Variable**	**Total (N=50)**	**Non Survivor (n=29)**	**Survivor (n=21)**	***P*** **-value**
Gender (Male)	29 (58%)	14 (48.3%)	15 (71.4%)	0.147
Age	57.2 ± 16.16	62.73 ± 17.74	49.4 ± 10.25	0.056
Limb paralysis	4 (8%)	3 (10.3%)	1 (4.8%)	0.556
syncope	9 (18%)	6 (20.7%)	3 (14.3%)	0.451
SBP (mmHg)	138.5 ± 27.5	140.69 ± 25.6	135.52 ± 30.4	0.780
Heart rate (Beats/min)	84.3 ± 2087	85.1 ± 23.62	83.38 ± 16.87	0.966
History of CAD*	11 (22%)	9 (31%)	2 (9.5%)	0.071
History of HTN*	31 (62%)	19 (65.5%)	12 (57.1%)	0.817
History of CVA*	3 (6%)	2 (6.9%)	1 (4.8%)	0.694
History of CKD*	1 (2%)	0 (0%)	1 (4.8%)	0.417
hs Troponin T level (ng/dl)	20.52 (9-100)	31.99 (16.3-125.8)	11.3 (5.6-40)	0.005
D-dimer level (ng/dl)	4.54 (1.2-106)	4.72 (1.7-7.7)	1.7 (0.6-19.6)	0.771
White blood cell count ( × 10³)	10.8 (9.3-13.3)	11.5 (9.7-13.7)	9.4 (8.7-13)	0.018
Platelet count ( × 10³)	173 (131.2-225.4)	152.1 (113-224)	197 (159.3-228.7)	0.771
Hemoglobin (mg/dl)	12.9 ± 2.25	12.54 ± 2.37	13.43 ± 2.01	0.241
Creatinine (mg/dl)	1 (0.8-1.5)	1.03 (0.8-1.6)	1 (0.8-1.25)	0.867
Ejection fraction (%)	53.2 ± 10.3	53 ± 10.26	54.4 ± 10.4	0.708
Moderate AI ≤	36 (73.5%)	22 (75.9%)	14 (70%)	0.588
Ascending aorta diameter (mm)	55.4 ± 14.5	54.4 ± 15.06	56.8 ± 13.9	0.431
Pericardial effusion	20 (40.8%)	12 (41.4%)	8 (40%)	0.855
Medical treatment	19 (38%)	17 (58.6)	2 (9.5%)	0.013
Surgical approach	31 (62%)	12 (44.4%)	19 (90.5%)	0.001

*SBP, Systolic blood pressure; CAD, Coronary artery disease; HTN, Hypertension; CVA, Cerebrovascular arrest; CKD, Chronic kidney disease; AI, Aortic insufficiency.

**Figure 3:**
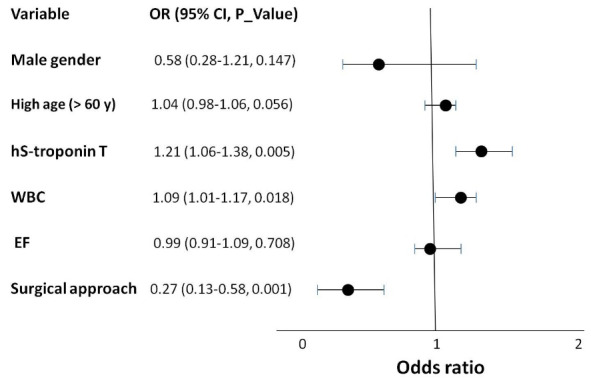
Forest plot of the odds ratio for 3 years mortality of acute aortic dissection type A. High hs-troponin T levels significantly increased long-term mortality. Also, long-term mortality was significantly less in the surgical approach

Multivariable analysis showed only an increase in hs-cTnT levels was suggested as a predictor of mortality (95% CI: 1.06–1.38; HR = 1.21; *P*= 0.005), so that for every 100 units increase, patients were 21% more likely to have mortality in long term. Also, performing surgical treatment for aortic dissection was determined as the independent predictor of surviving, so that death was 74.6% less than those who received medical treatment (95% CI: 0.13–0.58; HR = 0.27; *P*= 0.001) (Figure 3).

## Discussion

Therefore, AAD is a highly lethal medical or surgical emergency. However, to date, there is no clinical risk stratification model for patients with AAD^4^ and AAD is associated with a great deal of forgetfulness due to the lack of specific clinical symptoms.^5^ After clinical suspicion, its diagnosis is made by echocardiography or CT angiography. At present, none of the laboratory findings play with high sensitivity and specificity a role in the diagnosis and prognosis of patients.^6^ However, our study shows that hs-cTnT, WBC and NLR are significantly associated with long-term mortality in these patients. Other studies have shown prognostic role of D-dimer, C reactive protein (CRP), matrix metalloproteinases, smooth muscle myosin heavy chain, and soluble elastin fragments in AAD type A.^7^

Although the role of troponins in acute vascular syndromes including acute coronary syndromes and acute pulmonary embolism has been confirmed as a prognostic factor, its role in acute aortic dissection has not yet been well established.^8^ Troponin levels as seen in this study can be increased between 23% and 33% of patients with AAD type A which this condition increases the likelihood of misdiagnosis and missing of ADA. Mechanisms of increased Troponin levels in dissection include ischemia due to severe hypotension, ischemia due to severe aortic valve insufficiency, covering of the coronary artery by the intimal flap, and sometimes extension of the intimal flap into the coronary arteries.^8^ Most previous studies have focused more on increasing hs-cTn after surgery and its association with increased mortality,^9^ while surgery itself can increase hs-cTn and therefore have false results. Therefore, in this study, we included patients undergoing both medical and surgical treatment and examined preoperative hs-cTnT levels.^10^ Unlike to our study, the above studies and several other studies have also evaluated in-hospital and short-term mortality that increasing of hs-cTnT levels significantly increases mortality.^11^ However, the results of large sample of single center in China showed that an increase in troponin was not associated with long-term mortality of AAD. However, in the above study, hs-cTnI was examined, while in our study, hs-cTnT was considered.^12^ Except for the above study and our study; No other study has evaluated the association between long-term mortality and hs-cTnT.

The value of biomarkers depends on their clinical availability, and their cost-effectiveness is a precondition for their use in the clinical routine test.^13^ The utility of hs-cTnT measurement as a routine test for risk stratification in patients with AAD cannot be established by this single-center study and more large studies are required.

## Conclusion

 The results of present study showed the level of hs-cTnT has a potential utility for predict of long-term mortality in patients with AAD type A.

## Acknowledgements

 None.

## Competing Interests

 The authors declare that they have not competing financial interests or personal relationships that could have influenced the work reported in this paper.

## Ethical Approval

 This study was approved by ethics committee of Tehran heart center with the ethical code of 8751.

## Funding

 None.
